# Cation-Exchange Resin Applied to Paralytic Shellfish Toxins Depuration from Bivalves Exposed to *Gymnodinium catenatum*

**DOI:** 10.3390/foods12040768

**Published:** 2023-02-10

**Authors:** Joana F. Leal, Gabriel Bombo, Patrícia S. M. Amado, Hugo Pereira, Maria L. S. Cristiano

**Affiliations:** 1 Centre of Marine Sciences (CCMAR) and Department of Chemistry and Pharmacy, Faculty of Science and Technology, University of Algarve, Campus de Gambelas, 8005-139 Faro, Portugal; 2 GreenCoLab—Associação Oceano Verde, Universidade do Algarve, Campus de Gambelas, 8005-139 Faro, Portugal

**Keywords:** saxitoxins, *Gymnodinium catenatum*, mussels, depuration, cation-exchange resins, adsorption selectivity, structural effects

## Abstract

The accumulation of marine biotoxins in shellfish and their consumption causes serious food safety problems, threatening human health and compromising the availability of protein-based food. It is thus urgent to develop methodologies for the detoxification of live bivalves, avoiding their economic and nutritional devaluation. In this context, we tested an adsorption mechanism of paralytic shellfish toxins (PST) based on a cation-exchange resin. The first studies using cultures of *Gymnodinium catenatum* (natural producers of PST) showed a decrease of about 80% in overall toxicity after 48 h. Interestingly, we found that the toxins are adsorbed differently, with toxins’ structural features playing a part in the adsorption capacity via steric hindrance, electronic effects, or the extent of positive charge density (e.g., dcSTX). The positive effect of the resin in accelerating PST clearance from live mussels (*Mytilus edulis*) is not evident when compared to resin-free clearance; nevertheless, relevant information could be gathered that will facilitate further in vivo studies. Several factors appear to be at play, namely the competition of natural substances (e.g., salts, organic matter) for the same binding sites, the blocking of pores due to interactions between molecules, and/or difficulties in resin absorption by mussels. Additionally, the present work revealed the ability of mussels to neutralize pH and proposes bioconversion reactions among the PST molecules.

## 1. Introduction

**Figure 1:**
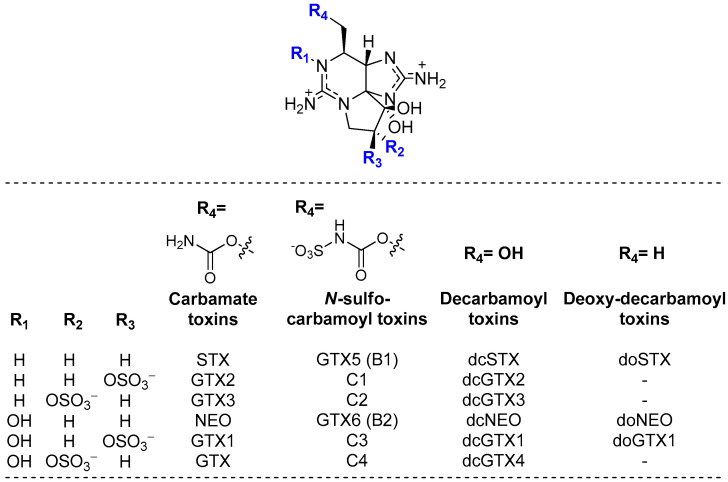
Structure representations of the major STX derivatives (PST group), with the positive charge fully delocalized over the pyrimidine and imidazole groups.

Paralytic shellfish poisoning toxins (PST) are a large group of highly potent neurotoxins and gonyautoxins produced by the harmful phytoplankton *Alexandrium*, *Gymonodinium*, and *Pyrodinium* [1]. The accumulation of PST in marine species such as mussels, clams, and fishes along the food chain causes a disease that could be lethal to marine mammals and humans [2,3]. The prevalence of paralytic shellfish poisoning (PSP) is reported annually along most of the world’s coastlines [4,5,6]. These toxins may potentially cause food poisoning and are associated to severe economic losses worldwide [5,7]. PST are a group of saxitoxin (STX) derivatives composed by about 50 analogues that have been reported in many organisms (Figure 1) [5,8]. Naturally occurring PST may be structurally modified by various biological factors.

In order to protect human consumption, depuration is a legal requirement for the marketing of fresh shellfish in several nations. Various strategies have been investigated for PST depuration in shellfish, involving chemical, biological, and physical procedures [9]. Among these, the application of adsorbents to eliminate PST appears to be promising, with different adsorbents already reported, such as chitin shell powder [10], clays [11,12], carrageenan [13], activated charcoal [14,15], and chitosan derivatives, either in the free form (chitosan) [16,17] or using carboxymethyl chitosan (CMC) [18].

Ion-exchange resins are polymeric granules or beads with several functional groups capable of binding ions with opposite charges [19]. They are either cation-exchange (CER) or anion-exchange resins, with CER found to adsorb positive charged compounds, including tricyclic antidepressants [20] and monosaccharides [21]. Despite the versatility and adaptability of cation-exchange resins, their interaction with PST has not been investigated.

PST are highly polar and hydrophilic molecules, due to the presence of a 3,4-propinoperhydropurine tricyclic system and two guanidine groups in their structure [22], and are mostly in the charged form at typical seawater pH [15]. Given the molecular structure of PST and their charged nature, together with their susceptibility of being adsorbed by different polymers and clays, we decided to explore the effect of a CER, specifically a styrene-divinylbenzene (ST-DVB) co-polymer bearing sulfonic acid functional groups, on the elimination of PST from cultures of *G. catenatum* and live bivalves contaminated over a 48-h period. PST were analysed using high-performance liquid chromatography with fluorescence detection (HPLC-FLD) and their profile was compared between matrices as well as throughout the adsorption process.

## 2. Materials and Methods

### 2.1. Growth of Toxic Microalgae

*Gymnodinium catenatum* IO13-26-02 was obtained from the ALISU culture collection (Lisbon University) and previously characterized regarding its toxin profile [23]. It was a PST-producing dinoflagellate that was grown under the following culture conditions: 19.0 ± 1.0 °C, under 14 h: 10 h light: dark cycle and 80 µmol m^−2^ s^−1^ light intensity. Cultures were grown with L1-medium [24], prepared with artificial seawater using sea salts (Classic Sea Salt, Tropic Marin) to a final concentration of 33 ppt, which was later sterilized at 121 °C and 15 psi for 20 min. To estimate cell concentration, samples were taken from concentrated cultures and fixed in Lugol’s acidified solution. The cell count was performed in quadruplicate using an inverted microscope (Zeiss Primovert, Carl Zeiss Microscopy GmbH, Jena, Germany) with 40× magnification on a Sedgewick–Rafter counting chamber [25].

In an early phase of the experiments, cultures of *G. catenatum* were used for the first studies of PST removal tests. For that, *G. catenatum* cultures were harvested at the end of the exponential phase with a concentration of 1.38 × 10^5^ cells. L^−1^. The culture was filtered using GF/C fiberglass filters 0.7 μm (Whatman^TM^, Cytiva, Global Life Sciences Solutions, USA LLC, Marlborough, MA, USA). The biomass was frozen at −80 °C, until its use. In subsequent experiments, *G. catenatum* cultures were prepared to feed live mussels. To this end, the cultures were grown in 6 L flat bottom balloon flasks [26], until concentrations in the order of 10^6^ cells. L^−1^ were reached.

### 2.2. Resins Preparation and Their Characterization

**Figure 2:**
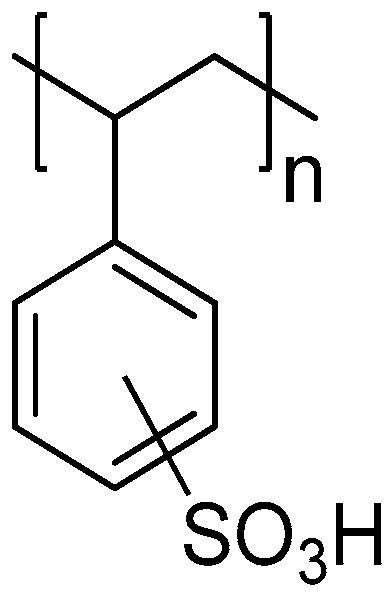
Structure representation of the acidic cation-exchange resin derived from the ST-DVB co-polymer.

To evaluate the ability to remove toxins in *G. catenatum* cultures and in contaminated bivalves, a strong acidic resin was used: Amberchrom^®^50WX8 hydrogen form (100–200 mesh, Thermo Fisher Scientific, Waltham, MA, USA, CAS 69011-20-7). It is a cation-exchange resin of styrene-divinylbenzene (ST-DVB) with sulfonic acid functional groups as aryl substituents (Figure 2). For studies of toxin removal in culture and in bivalves, the resin concentration was 1 g/L.

Before its use, the resin was cleared and activated as follows [27]. Deionized water—10 times by volume (mL) of the resin mass value (g)—was added to the resin, in a beaker. The mixture was stirred with a glass rod and allowed to settle for 20 min. Then, the fine particles were decanted. To activate the resin, the same volume of 1 M H_2_SO_4_ (95%, Fisher Chemical, Fisher Scientific UK, Loughborough, UK) was added, and the mixture was left for 15–20 min, after which the fine particles were again decanted. This activation process was repeated three times. Then, the resin was rinsed with deionized water until pH > 5, using a Büchner funnel and PVDF membrane filter paper 43–48 µm (Filter-Lab, Filtros Anoia S.A., Barcelona, Spain). Additionally, a sodium-form (Na^+^-form) of the resin was prepared from the acidic form (H-form) as follows: deionized water—10 times by volume (mL) of the resin mass value (g)—was added to the resin in a beaker. The mixture was stirred with a glass rod and was allowed to settle for 20 min. Then, the fine particles were decanted. To convert the resin to the sodium form, the same volume of 4% NaOH (p.a., Chem-Lab NV, Zedelgem, Belgium) was added, and the mixture was left for 15–20 min, after which the fine particles were again decanted. Then, a solution of 4% NaCl (p.a., ≥99.8 %, Chem-Lab) was added, the mixture was left for 15–20 min and later decanted. This final step was repeated twice. The resin was then rinsed with deionized water until pH = 7, using a Büchner funnel and PVDF membrane filter paper 43–48 µm (Filter-Lab). Both forms of the resin (H and Na^+^) were dried in an oven at 50 °C for further characterization by Fourier Transform Infrared Spectroscopy (FTIR), X-ray diffraction (XRD), and optical microscopy (OM).

Powder X-ray diffractograms were recorded on a PANalytical X′Pert Pro diffractometer using Cu Kα radiation (λ = 0.15418 nm) filtered by Ni and an X’Celerator detector, operating at 50 kV and 30 mA. The patterns of the resins were recorded in the range 3–70° (2*q*), with a step size of 0.334225° and 400 s per step. Plots were generated with manual R scripts in RStudio (Version 2022.07.2), using ggplot2 libraries for the graphic figures.

FTIR tests were performed to analyse the functional groups present in the resins. The samples were previously grounded with KBr in a mortar and compressed into discs. FTIR spectra were recorded on an FTIR spectrophotometer (Bruker, model Tensor 27, Massachusetts, USA). For each spectrum, a 25-scan interferogram was collected in transmittance mode in the 4000–600 cm^−1^ region, with a 4 cm^−1^ resolution. The results were obtained using the OPUS software (V6.5, Bruker Optik GmbH 2009, Ettlingen, Germany). Plots were generated with Microsoft Excel (Microsoft 365 Apps).

To evaluate particle shape and size, an optical microscope (Microscope Zeiss ESTATIVO AXIOSCOPE 5-TL/FL6XH DIC, coupled with Zeiss Axiocam 305 (Carl Zeiss Microscopy GmbH, Jena, Germany) with particle diameter measurement on the Zen Blue Image processing software was used.

### 2.3. Toxin Removal Studies Using Culture of G. catenatum

PST removal tests were first performed on *G. catenatum* cultures to understand the effect of the resin on each analogue. For this, PST were extracted from the culture as follows: 3 mL of 1% acetic acid (prepared from glacial acetic acid, HPLC grade ≥ 99.8%, from Carlo Erba reagents S.A.S, Val de Reuil Cedex, France) was added to a falcon tube containing the fibreglass filter with biomass. The filter was crushed with a metal lancet [23] to enhance the extraction yield, and the mixture was stirred using a vortex (±10 s). The tubes containing the samples were placed in the ultrasounds bath (Transsonic 660/H Elma Schmidbauer GmbH, Singen, Germany), with ice, for 10 min. Thereafter, the tubes were placed in a water bath at 100 °C for 5 min, vortexed again (±10 s) and placed in a beaker with ice for 5 min. The samples were centrifuged at a low temperature (10 °C) for 10 min at 3600 *g*, using a high-speed centrifuge (Eppendorf 5430 R, Eppendorf SE, Hamburg, Germany). The supernatant was filtered into a flask, using PVDF filters 0.22 µm, 13 mm (Teknokroma, Barcelona, Spain). The addition of 1% acetic acid (3 mL), vortexing, centrifugation, and filtration of the supernatant into the same flask were repeated twice. The blank was subjected to the same experimental procedures as the samples.

Afterward, the solution of PST (extracted from the culture) was added to beakers containing the previously weighed resin, together with small magnetic bars. The beakers were distributed on a multi-position magnetic stir plate (2mag, Germany), in which the mixtures were stirred simultaneously at 120 rpm for five different times: 1, 3, 19, 26, and 48 h. Additionally, controls (toxins without resin) were performed at 0 and 48 h. At the end of each time, all samples were immediately filtered into a glass vial, using syringes and PVDF filters 0.45 µm, 13 mm (Teknokroma). Four replicates of these experiments were carried out.

### 2.4. Toxin Removal Studies in Live Bivalves

In this work, three sets of experiments were carried out using live mussels (*Mytilus edulis*): (1) with the H-form resin and healthy mussels; (2) with the H-form resin and mussels contaminated with *G. catenatum*; (3) with the Na^+^-form resin and mussels contaminated with *G. catenatum*. All tanks had continuous aeration and a small aquarium pump (3 W) was used to increase water flow. All mussels used in these experiments were harvested in Ria Formosa, Algarve (southern Portugal), depurated (≥48 h) and transported in thermal containers from the depuration centre to the laboratory (approximately 20 min). In addition to the mussels used in the feeding and depuration protocols, uncontaminated mussels were collected in each set of studies to subsequently evaluate the matrix effect in the quantification method.

Four small tanks were filled with 10 L of seawater (salinity: 37.86 ± 0.02), prior to the feeding experiments with toxic microalgae and the addition of resin, to assess the purification process’s acceleration. At this stage, ten previously purified mussels were randomly distributed in each tank, with aeration, and allowed to condition for approximately 19 h. Following this period H-form resin was added to two tanks, while the other two remained resin-free. The objectives of this first set of experiments were to investigate possible signs of acute toxicity of the resin for the mussels studied and to assess the mussels’ ability to ingest the resin. Observations were carried out at 24 and 48 h. After each time, the mussels, exposed or not to the resin, were removed from the tanks, washed outside with fresh water, then opened to analyse their digestive tract. The mussels used in this study had an average size of 6.2 (±0.6) × 3.9 (±0.3) cm.

**Figure 3:**
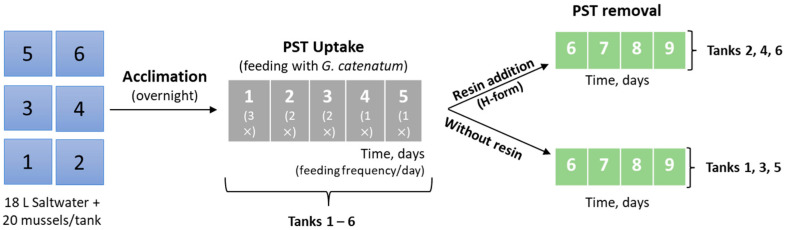
Experimental design for the feeding and clearance of live bivalves (second set of experiments). The numbers in the boxes on the left correspond to the tanks while the others (in the middle and on the right) correspond to the number of days.

Since the preliminary test revealed no signs of acute toxicity (no mortality), a second set of experiments was performed in triplicate, using six rectangular tanks with 18 L of saltwater each. The experimental design is illustrated in Figure 3. Twenty live mussels were randomly placed in each tank. The average size of mussels in each tank is shown in the Supporting Information, SI (Table S1). The mussels were acclimatized to the new medium overnight (about 18 h) and there was no mortality throughout this period.

**Figure 4:**
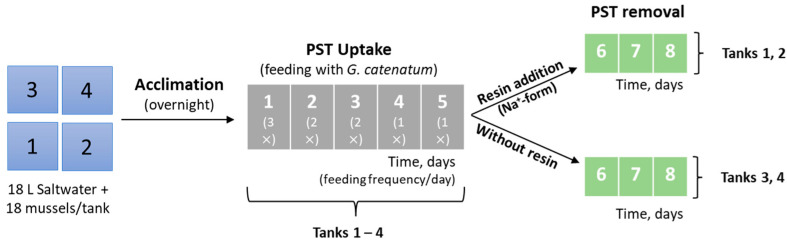
Experimental design for the feeding and clearance of live bivalves (third set of experiments). The numbers in the boxes on the left correspond to the tanks while the others (in the middle and on the right) correspond to the number of days.

The third set of experiments (Figure 4) was carried out under similar conditions as the second. Briefly, 18 mussels were placed in each tank containing 18 L of saltwater. Acclimation occurred overnight, followed by feeding of *G. catenatum* as described below. Table S2 shows the average size of the mussels used in this experiment. These experiments were performed in duplicate and no mortality was observed during the entire experiment.

Several physicochemical parameters (percentage of dissolved oxygen, % DO; temperature, T °C; salinity; pH) were regularly monitored throughout the experiments with live mussels, using a multiparameter (HI98194, HANNA Instruments, Cluj Napoca, Romania) and the corresponding probes. DO remained over 70% throughout the experiments. Laboratory biosafety guidelines were considered for all experiments involving toxic microalgae and live bivalves to avoid human and/or environmental contamination.

#### 2.4.1. Feeding with Toxic Microalgae

After the acclimation period, the algae culture was added directly to tanks containing live bivalves, for the second and third sets of studies, simulating a red tide bloom. The feeding with approximately 90,000 cells/bivalve/day occurred for five consecutive days [28]. The toxic food was given with the following frequency: 3 times on day 1, twice on days 2 and 3, and once on days 4 and 5. During the experiment, suspended material was removed and the water was partially renewed daily, before feeding. The averages (±standard deviation, SD) of temperature, salinity, and pH throughout the second set of experiments were 19.6 ± 1.8 °C, 36.1 ± 0.9 and 8.0 ± 0.2, respectively. Similar average values were obtained in the third set of experiments: 17.2 ± 0.8 °C, 36.6 ± 0.4 and 7.9 ± 0.1, for temperature, salinity, and pH, respectively. In the second set of experiments, nine of the hundred and twenty mussels died over the five days (one mussel in tanks 1, 4, and 6; three mussels in tanks 2 and 3). They were removed from the tanks as soon as detected, to avoid contaminations. In the third set of experiments no mortality was observed throughout. This situation might be partially explained by the fresher mussels and lower water temperature in the third set of experiments, compared to the second set.

Water samples were obtained from each tank 24 h after the last toxic food administration and fixed in Lugol’s acidified solution to determine the cell concentration of *G. catenatum*. Moreover, at the same time (day 6), 4–5 mussels were harvested in each tank for later determination of PST concentration. These determinations will correspond to time 0 (T0), when maximum mussel contamination is assumed. Furthermore, samples of water from each tank were obtained immediately at the onset of the depuration phase, to verify any remaining microalgae that was not consumed by the bivalves. 50 mL of sample from each tank were sedimented on a Utermöhl sedimentation chamber and observed on an inverted microscope (Zeiss Primovert, Carl Zeiss Microscopy GmbH, Jena, Germany) with 40× magnification.

#### 2.4.2. PST Depuration

At the start of the depuration phase, around 50% of the saltwater was renewed in each tank. Then, the mussels were submitted to a 70 h (second set) or 48 h (third set) clearance period. In tanks without resin, purification occurred naturally, without feeding. To the remaining tanks, the cation-exchange resin (H-form or Na^+^-form, 1 g/L) was added to assess its ability to accelerate PST clearance from bivalves. As expected, the resin remained suspended in water due to its properties. 3–5 mussels from each tank were collected after 24 h (time 1, T1) and 48 h (time 2, T2), in both sets of experiments, with additional sampling for the second set after 70 h (time 3, T3). These periods were selected since they are like those used in bivalve purification facilities [29]. Some mortality occurred during depuration in the second set, with a loss of two mussels from tank 2: one before T1 and the other at T3. No mortality was detected in the other tanks. Physicochemical parameters (% DO, T °C, and pH) were measured regularly during these experiments.

After collecting the mussels, their treatment started for up to 2 h. As described in the AOAC Official Method 2005.06 and its standard operating procedure [30,31], the mussels were washed with cold water and left to dry. Afterward the shell was opened, all tissue was removed and placed in a sieve to drain. The tissue was then homogenized with an ultra-turrax (Janke & Kunkel GmbH & Co.KG IKA^®^-Werke, Staufen, Germany), and 5.0 ± 0.1 g of each sample tissue was weighed into 50 mL polypropylene centrifuge tubes. The remaining extraction steps were the same as described in the AOAC Official Method 2005.06.

### 2.5. Clean-Up, Fractioning, and Pre-Oxidation of Samples

For clean-up and fractionation of the samples solid-phase extraction (SPE) was applied, using C18 (500 mg/3 mL, Finisterre, Teknokroma, Barcelona, Spain) and COOH (500 mg/3 mL, Speed^TM^ Applied Separations, Allentown, PA, USA) cartridges, respectively. For mussels, the adopted procedures of SPE-C18 and SPE-COOH were the same described in the AOAC Official Method 2005.06. After SPE-C18, all samples from mussels were passed by PVDF filters 0.45 µm.

For samples from *G. catenatum* cultures, the SPE-C18 procedure applied was adapted from the official method, with the adaptations highlighted as follows. After conditioning, 2.5 mL of each sample or blank were added to each cartridge. An amount of 2.0 mL of ultrapure water was added to wash the cartridge, and the final volume was adjusted to 5.0 mL (after pH adjustment to 6.5) [23]. The SPE-COOH procedure was the same as in the official method. In all these procedures, ultrapure water (Simplicity^®^ Water Purification System, Merck KGaA, Darmstadt, Germany, 18.2 MΩ.cm) and analytical or HPLC grade solvents were used. After SPE, the samples were stored at −18 °C until oxidation for subsequent analysis.

Before analysing the PST from the different matrices, an additional pre-oxidation step was necessary to allow their determination by HPLC-FLD. The samples from SPE-C18 were subjected to oxidation with peroxide (specific to detect non-*N*-hydroxylated toxins) and periodate (able to detect *N*-hydroxylated and non-*N*- hydroxylated toxins). The samples from SPE-COOH were only submitted to periodate oxidation.

The peroxide and the periodate oxidation procedures for bivalve samples were those described in the official method [30,31]. Matrix modifier was prepared from oysters *Crassostrea gigas* harvested in Ria Formosa, Algarve (southern Portugal), which were previously depurated and submitted to the same procedures of preparation, extraction and SPE-C18 clean-up described above for mussels. Oysters’ extracts were stored in a freezer (−18 °C). In turn, the periodate oxidation procedure for samples from culture had minor modifications. Instead of the matrix modifier, ultrapure water was used, as suggested by other authors [32]. More details about this procedure and the conditions of preparation of the oxidizing agents may be found in our previous publication [23]. In both procedures, the periodate oxidant was freshly prepared on each day of analysis.

### 2.6. HPLC-FLD Analysis

An HPLC-FLD from Shimadzu (Prominence-i LC-2030C Plus) was used to conduct the PST analyses. This equipment comprises a quaternary pump, a refrigerator autosampler, a column oven and a spectrofluorometric detector (RF-20A XS). The temperatures used were 10 °C, 25 °C and 30 °C, in the autosampler, column oven and cell detector, respectively. A reversed-phase C18 column (Mediterranea Sea18, Teknokroma), 25 cm × 0.46 cm (5 µm), and an ultraguard^TM^ column (Sea18 10 × 3.2 mm, Teknokroma) were used. Ammonium formate 0.1 M (adjusted to pH 6 with acetic acid 0.1 M) and acetonitrile (HPLC grade, Honeywell) were applied as mobile phase A and B, correspondingly. All eluents and solvents for HPLC were filtered, using PVDF membrane filters (0.22 μm, 47 mm, Teknokroma). The gradient elution conditions were as follows. First 6 min: 1–5% phase B; between 6 and 13 min: 5–28% phase B; between 13 and 16 min: 28–1% phase B; between 16 and 19 min: 1% phase B. The flow rate was 1.5 mL/min. The injected volume was 30 µL or 100 µL, for standards and samples oxidised with peroxide or periodate, respectively. Three injections of each sample were performed. Fluorescence detection was achieved at 340 nm (excitation) and 395 nm (emission).

To quantify PST, calibration solutions ranging 0.03 and 2.00 µM were prepared from certified reference materials (CRM): dcGTX2&3, C1&2, GTX2&3, GTX5 (or B1), GTX6 (or B2), GTX1&4, C3&4, NEO, dcNEO, STX (from CIFGA laboratory S.A., Lugo, Spain) and dcSTX (from National Research Council of Canada, Halifax, Canada). To prepare the solutions, the following mixtures were considered: (I) dcGTX2&3, C1&2 and dcSTX (for peroxide oxidation); (II) GTX2&3, GTX5 and STX (for peroxide oxidation); (III) GTX1,4 and NEO (for periodate oxidation); (IV) C3&4 and GTX6 (for periodate oxidation); (V) dcNEO (for periodate oxidation); (VI) dcGTX2&3 and dcSTX (for periodate oxidation). The quantification of each toxin in the samples was performed by interpolation of data in the corresponding calibration curve. The contribution of the chromatographic peaks corresponding to the matrix modifier were subtracted (before interpolation) from the chromatograms of the samples and standards pre-oxidized with periodate in the presence of the matrix modifier. The limits of detection and quantification (LOD and LOQ) were determined as (3σ)/m and (10*σ*)/m, where σ is the residual standard deviation of the regression line and m is the slope of the calibration curve [33,34]. For the several calibration curves, all determination coefficients (*R^2^*) were above 0.9981. LOD and LOQ ranged from 0.003 µM (1 µg STX.2HCl eqv/Kg) to 0.03 µM (169 µg STX.2HCl eqv/Kg) and from 0.01 µM (3 µg STX.2HCl eqv/Kg) to 0.11 µM (615 µg STX.2HCl eqv/Kg), respectively (Table S3). The least concentrated standard solution of each calibration curve was always equal to or greater than the corresponding LOQ. To avoid misinterpretation, the following solutions were also analysed under the same conditions as the standards and samples: a chemical blank of the entire procedure—extraction, cleaning, fractionation, and oxidation (1% acetic acid), samples without the oxidizing agent (using the same volume of ultrapure water instead of the peroxide or periodate oxidant), the matrix modifier oxidised with periodate (the same volume of ultrapure water instead the sample/standard) (some examples in SI, Figures S1–S3).

### 2.7. Statistical Analysis

Data were calculated as the mean of at least three independent replicates ± standard deviation (SD). When suspected values were obtained, Dixon’s test was applied to test whether it was an outlier. To check whether the variances are different or not, the *F*-test was applied. Then, Student’s *t*-test was used to assess for differences between the averages of two sets of results. For both statistical parameters, the significance level considered was 0.05. The results are considered statistically different when the calculated values exceed the critical values [34]. The statistical treatment was performed with Microsoft^®^ Excel^®^.

## 3. Results and Discussion

### 3.1. Characterization of the Resins

#### 3.1.1. X-ray Diffraction (XRD)

**Figure 5:**
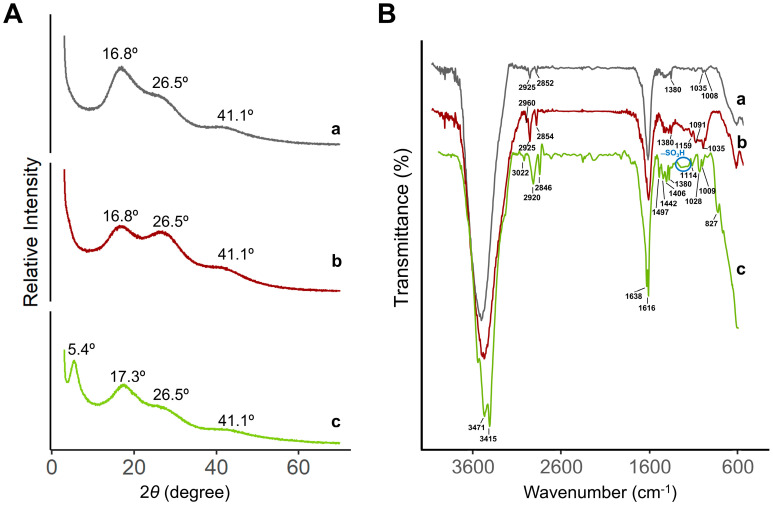
(**A**) XRD patterns and (**B**) FT-IR spectra of resins: (**a**) inactivated H-form, (**b**) activated H^+^-form, and (**c**) Na^+^-form.

Figure 5A depicts the XRD patterns of the various resins, the H-form (activated and inactivated) and the Na^+^-form. All patterns exhibit a substantial amorphous area at angles above 40°. Inactivated and activated H-form resins show two large and broad crystalline zones at angles of 16.8° and 26.5°, although with differences in the relative intensities for the angles. The inactivated form has a greater intensity at 16.8°, whereas the activated H-form has a greater intensity at 26.5°. This observation suggests that saturation/activation with H^+^ somewhat modifies the crystalline structure of activated versus non-activated resin. At an angle of 5.4°, a new sharper peak is visible for the Na^+^-resin. Notably, the broad peak previously seen at an angle of 16.8° in resins of the H-type (activated or inactivated) is slightly shifted to an angle of 17.3°. The peak at 26.5° is still detectable, although residual. The obtained results with the Na^+^-form resin indicate that the crystalline structure of ion-exchange resins is highly dependent on the cation with which they interact. To the best of our knowledge, the characterization of these resins by XRD has not yet been described in the literature.

#### 3.1.2. Fourier Transform Infrared Spectroscopy (FTIR)

The FTIR spectra of activated/inactivated H-form and Na^+^-form resins were measured in the of 4000–600 cm^−1^ wavenumber range (Figure 5B). The characteristic peaks for styrene-divinylbenzene linkages in the resin structure were observed in all resins. Peaks at 3022 cm^−1^ and 2920 cm^−1^ correspond to the C-H bonds in the benzene ring and -CH_2_ in the cross-linked polystyrene matrix. The sharp peaks at 1638 cm^−1^ and 1616 cm^−1^ are representative of the C=C– bond vibrations of the styrene system [35]. Bands at 3471 cm^−1^ and 3415 cm^−1^ were characteristic of stretching vibrations of O-H bonds and were broader in the H-form resins. Peaks at 1497, 1442, 1406, and 1380 cm^−1^ correspond to the stretching and bending of aromatic –CH– bonds of the ST-DVB, respectively [36]. The broad peak between 1166 and 1250 cm^−1^ corresponds to the distinctive absorption peaks of the functional sulfonic acid groups (–SO_3_H) [37]. Furthermore, the peak band between 827 cm^−1^ and 1114 cm^−1^ may correspond to the benzene ring stretching vibrations induced by the resin’s ST-DVB link matrix [35]. Signals in the H-form resins (both activated and inactivated) appeared to be less resolved, probably due to the higher water retention capacity, comparing to the Na^+^-form.

#### 3.1.3. Optical Microscopy

**Figure 6:**
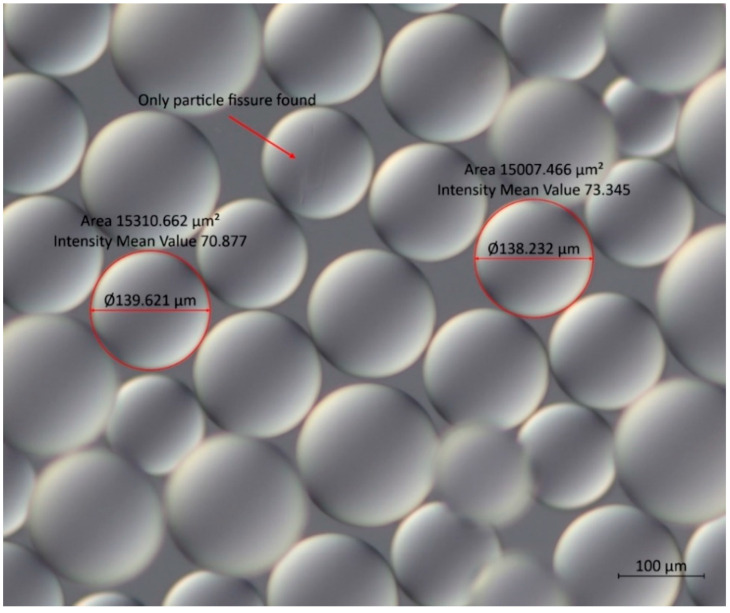
Microscopic image with DIC filter from particles of the Na+-form, showing the only deformation observed and two samples of the measurement taken from the particles.

In the Na^+^-form, the resin showed spherical forms on a microscopic view (100× magnification), with average particle diameters of 140 ± 12 µm (*n* = 100, Figure 6). The spheres show almost no fissures, with a 1% ratio of fissures on the sampled resin particles. For the H-form, the average diameter of the particles was 155 ± 19 µm, showing no damage in the particles observed. This implies that the H-form has a bigger variation in size and shows better stability regarding the form; on the contrary, the Na^+^-form particles show better stability concerning the particle size but show imperfections in the formation of the particle. When comparing both resins, the variation in size, however minor, may facilitate the adsorption of the toxin or even its passing through the bivalve’s digestive system, indicating that the Na^+^-form properties could be more promising to promote the depuration. The particles of both types of resin are translucent, showing a yellow colour to the naked eye that is not visible under a microscope.

### 3.2. PST Removal from G. catenatum Cultures

Along the experiment, that lasted for 48h, the toxins GTX2&3 and STX were not detected, while the toxins dcGTX2&3, C3&4, GTX1&4, NEO, and dcNEO were detected at levels below the corresponding LOQ (for all replicates). On the other hand, the toxins C1&2, dcSTX, GTX5 and GTX6 were quantified. The results of the PST removal experiments over 48 h with the H-form resin are shown in Table 1. For toxins C1&2 and GTX6, no changes in concentration were observed throughout the experiment. However, after 48 h of contact with the H-form resin we obtained an average removal of 55% and 100% of the concentration of GTX5 and dcSTX, respectively. The removal percentage was determined following Equation (1), where *C_i_* is the concentration for each toxin. These are very promising results, especially due to the complete removal of dcSTX, which has a high toxic potential.

Considering the toxic potential of each toxin (proposed by EFSA) and calculating the total toxicity equivalent to saxitoxin (Equation (2)), a decrease in the total toxicity close to 80% is obtained after 48 h of contact with the resin (relative to the same period without resin—48 h control). No differences were observed between the zero-time experiment (before addition of resin) and the 48-h control (with toxins but without resin), this meaning that there was no degradation of the toxins along the experiment. As such, we can safely conclude that the observed decrease in the concentrations of GTX5 and dcSTX along the 48 h was due to chemical adsorption to the H-form resin.

(1) $$\%\;Removal_{48h}=(1-\left(C_{i,\;48\;h}/C_{i,\;0\;h})\right)\times 100$$

(2) $$C_{total}\left(\mu M\right)=\sum TEF_{i}\times C_{i}$$

**Figure 7:**
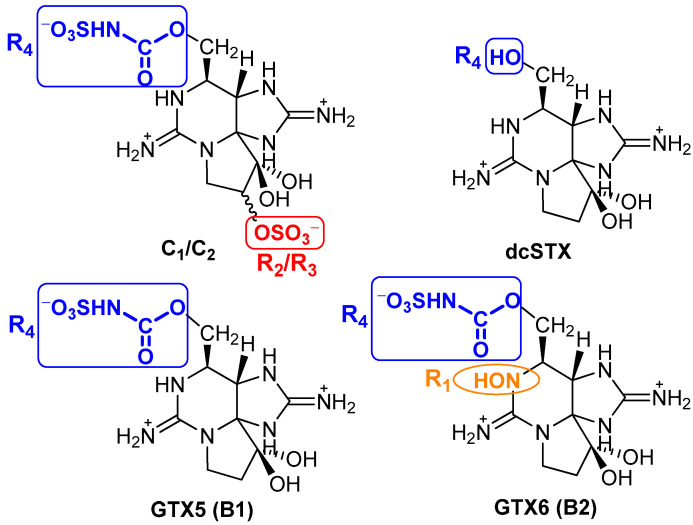
Molecular structures of some PST, under acidic conditions [2].

To better understand the selectivity/specificity of a given type of reaction over another, here expressed by the differences in affinity to the resin evidenced by the various quantified toxins, Figure 7 shows a representation of their molecular structures. Unlike the C1&2, GTX5 and GTX6 toxins, the decarbamoyl toxin dcSTX bears a hydroxyl group (-OH) at R_4_, which renders it less sterically hindered than the other toxins (all of which contain an *N*-sulfocarbamoyl group, -OCONHSO_3_^−^, at R_4_). This steric effect, combined with the overall positive charge, will probably facilitate the interaction of the toxin with the resin structure, promoting an easier adsorption through exchange of H^+^. In addition to the *N*-sulfocarbamoyl group on R_4_, toxins C1&2 and GTX6 differ from dcSTX and GTX5 in other functional groups: C1&2 bear an *O*-sulphate group on R_2_/R_3_, while GTX6 bears an -OH group in R_1_. The presence of the *O*-sulphate group contributes to an even greater steric impediment and affects the charge balance (neutralizes), disfavouring the exchange of cations with the resin. In turn, the -OH group in GTX6 introduces the additional effect of electronegativity, disfavouring interactions with the resin through electronic effects. In fact, a similar behaviour was observed by other authors [38]. These authors tested the adsorption of eleven PST to magnetic nanoparticles. From the experiments, they reported a very low removal for the toxins C1 and GTXs (GTX1-6 and dcGTX2-3), in contrast with STX, dcSTX, NEO, and dcNEO, attributing the low adsorption/removal of those toxins to the presence of the sulphate side groups in their structure.

### 3.3. PST Removal from Bivalves

**Figure 8:**
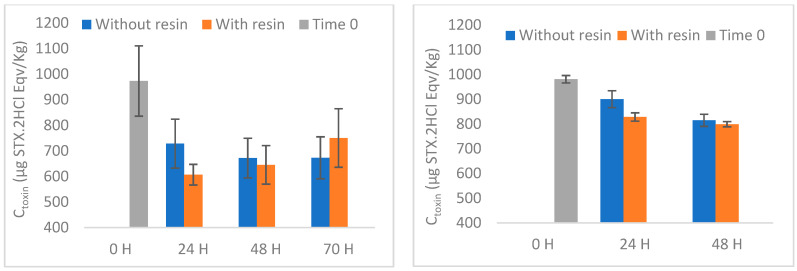
PST clearance results (global values) from live bivalves using H-form resin (**left**) and Na^+^-form resin (**right**). Natural clearance (without resin) is also presented for each set of experiments.

Boosted by the previous encouraging results, the toxin removal experiments were replicated (with appropriate adjustments) on live mussels. On day 6, immediately before adding the resin, the collected water samples did not show any cells of *G. catenatum* or cysts of the algae, implying that the bivalves on the tanks consumed all available algae. Similarly, the HPLC-FLD analysis of toxins from water samples collected from different reservoirs revealed no quantifiable toxins. For those reasons, the toxin concentration at time 0 (day 6) is assumed as the starting point for assessing mussel PST clearance. In both experiments involving contaminated mussels by *G. catenatum*, the toxins GTX2&3 and STX were undetectable throughout all experiments. The toxins C3&4, GTX1&4 and NEO were not quantifiable over the course of the experiments. dcNEO was also unquantifiable at time zero and undetectable at the remaining times. In the second set of experiments, dcGTX2&3 was only quantified in tank 1 at zero time (34 µg STX.2HCl eqv/Kg) and after 24 h, without resin (24 µg STX.2HCl eqv/Kg). In the remaining tanks and times, it was unquantifiable. The results of the PST clearance from mussels, with or without resin, are shown in Figure 8, Figures S4 and S5 (individually) and Table 2.

#### 3.3.1. Time 0 and Natural Depuration

The toxin profile of *G. catenatum* determined in this work (Section 3.2) was considered for comparison with the toxin profile in bivalves. For a better comparison, an estimate of the culture concentrations (in µg STX.2HCl eqv/Kg) is presented in Table S4. It is considered that the toxins quantified in mussels are the same as in culture, in agreement with previously reported results using similar conditions [28]. However, there are differences in the proportion of toxins between the different matrices (culture vs. bivalves). The general increase in the amount of toxins may be partially justified by the higher concentration of cells offered as food to the mussels, compared to the concentration used in the removal experiments in cultures. However, we observed that the ratio between the toxins quantified in mussels and in culture varies for each of them (C1&2 = 1.4, dcSTX = 3.4, GTX5 = 2.7, GTX6 = 2.0), suggesting that biotransformations occur in live mussels. In fact, several bioconversions among toxins in live bivalves have been reported [28,39,40,41,42,43,44,45,46,47,48]. Note that, for example, C1&2 and GTX5 have the same TEF [49] and dilution factor, so they would be similarly affected if there were no other factors involved. However, a higher production of GTX5 than C1,2 is observed, which is interesting since C1,2 toxins may generate GTX5 through reductive cleavage of the *O*-sulphate group at C11 [41]. At the same time, the possibility of GTX5 oxidation at N1, originating GTX6, is not ruled out, since this type of reaction has already been reported in mussels *Mytilus* spp. [39,45]. Based on previously compiled information [8], dcSTX could be formed from STX, GTX5 or GC3 (in all cases by hydrolysis). In the present work, STX was not quantifiable and GTX5 seems more likely to be involved in other reactions. On the other hand, the higher abundance of GC toxins (hydroxybenzoyl derivatives) produced by *G. catenatum* has been reported [28,50]. In the present work GC toxins were not quantified, since LC-MS is a more appropriate method for this. Nevertheless, some authors have reported a higher abundance of dcSTX in bivalves, to the detriment of a decrease in GC3, contrary to what was observed in *G. catenatum* (higher relative proportion of analogous GC) [28,51]. This suggests conversion of GC3 to dcSTX through hydrolysis involving the R_4_ group. This reaction has been attributed to the enzymatic activity of a carbamoylase, targeting this subgroup. Nonetheless, the possibility of converting GC3 into dcSTX under the acidic conditions of the extraction process (acid hydrolysis) should not be disregarded [51].

In our experiments, after 5 days of feeding with *G. catenatum* all mussels analysed revealed cumulative levels of toxicity higher than those legally established—800 µg STX.2HCl eqv/Kg [49]. Similar values for the total toxicity were obtained in both sets of experiments, with mean values of 973 and 981 µg STX.2HCl eqv/Kg. The results from natural depuration experiments suggest faster clearance in the second set of experiments than in the third, corresponding to 33 ± 11% and 16 ± 3%, respectively, after 48 h. Another observation was a greater variation of the results in the second set, compared to the third set. Several conditions may contribute to this, and it is practically impossible to control all of them, mainly because the experiments involved live bivalves. In addition to the conditions inherent to the mussels used in each set of experiments (e.g., physiology and well-being), there are other parameters that may affect the efficiency of purification, namely temperature, salinity, oxygen levels and the particles suspended in water [29]. *M. edulis* are classified as rapid detoxifiers, the faster clearance observed in an initial phase being commonly attributed to the elimination of toxins not yet assimilated and incorporated by the tissues [48,52].

#### 3.3.2. Depuration with H-Form and Na^+^-Form Resins

As the identified toxins are mostly positively charged, a cation-exchange resin was used to try to accelerate the purification process. When using the H-form resin, an acceleration of the average clearance rate seems to occur after 24 h, mostly attributed to the decrease in dcSTX concentration (Figure S4). However, due to the large variation observed, this increase in acceleration is not statistically significant (*p* = 0.056). At 48 and 70 h no significant changes are observed, which could be related to the typical deceleration of clearance after unassimilated toxins have been released [52]. Several factors may contribute for the low effectiveness of the resin tested, namely absorption of the resin by the mussel and the presence of other water constituents (e.g., cations in saltwater, organic matter). Seawater contains various cations with different selectivity for this resin [53], which may compete with toxins for the same binding sites. Moreover, organic matter (OM) may affect the adsorption, the effect being variable with pH [15]. Three arguments have been used to explain the reduced adsorption of PST in the presence of OM, namely, pore-blocking, preferential interaction/competition by the active sites available, and the electrostatic interactions with PST [54]. Regarding the absorptive capacity of mussels, we did not observe large amounts of resin inside their digestive tract in preliminary experiments carried out (first set of experiments). Interestingly, the highest number of particles was observed in the digestive tract of the mussel after 24 h, while after 48 h few particles were observed. This could mean that the resin particles are not truly absorbed by the mussel, which renders the action of the resin more difficult. Contrary to what happened during the experiments in water, with toxins coming directly from the culture, in the resin experiments with bivalves there is no direct interaction between the resin and the PST absorbed by mussels.

**Figure 9:**
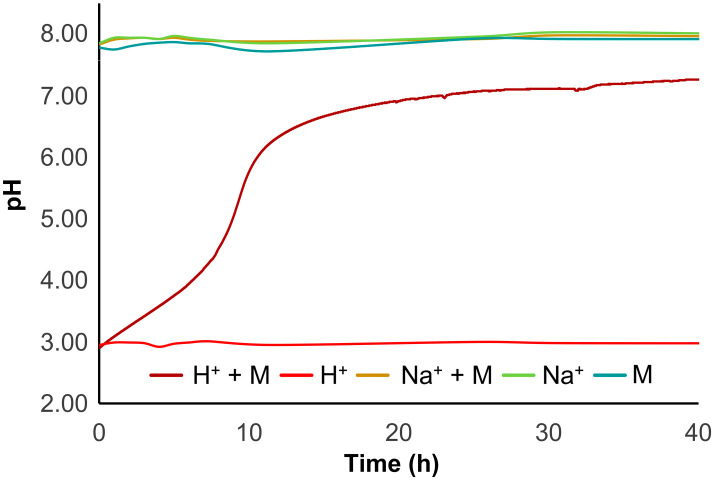
pH effect upon addition of the different resins, and respective controls.

During the experiments with H-form resin, an interesting behaviour was observed: a sudden pH oscillation was registered after the addition of the H-form resin, stabilizing until the end of the experiment. To better understand what happened, a parallel in vivo experiment was carried out. To three small containers with 2 L of saltwater was added, separately: (1) H-resin (Control 1); (2) H-resin with two mussels (H^+^ + M); and (3) two mussels, as a negative control group (M) (Figure S6). All containers had continuous aeration and a small aquarium pump was used to increase water movement. The pH was measured regularly along a period of 40 h and the results are depicted in Figure 9. The negative control group (container 3) displayed pH values in the ranges 7.75–7.93. Immediately after adding the H-resin to the containers 1 and 2 the pH decreased to 3.00. The pH in the H-resin container (container 1) remained constant over time, but the pH in the container with H-resin and mussels (container 2) gradually increased. The pH rise occurred as a steady acid–base titration, with a strong increase after 8 h and reaching a plateau at 24 h, with a pH close to 7. After 40 h, the pH reached a maximum of 7.26. This result suggests the shellfish’s role in adaptability during depuration in acidic conditions. The mussel shell is composed of calcium carbonate, CaCO_3_, and it would have been the reaction between CO_3_^2-^ and H^+^ in the acidic aqueous medium that would have promoted the pH increase [55] in the container with mussels, unlike for the container without mussels. Although the mussels bear the ability to adjust the pH of the medium, this excessive acidification is likely to affect not only their shell, but also their well-being [56]. Moreover, due to acidification, complete closure of mussel shells was observed in tanks where resin was added. This defensive response by the organisms will likely have inhibited their clearance rate during the first hours. Therefore, we think that the H-form resin, under the conditions in which it was prepared, may not be the most suitable matrix for in vivo studies involving bivalves.

Aiming to overcome the acidification observed previously, the resin was modified by converting the H-form into the corresponding Na^+^-form. First, in a parallel experiment to the one described above, two additional conditions were tested: (4) Na^+^-resin (Control (2) and (5) Na^+^-resin with two mussels (Na^+^ + M) (Figure S6). When using the Na^+^-form resin, after introducing the resin to the tank with shellfish the pH remained steady, ranging between 7.83 and 7.98. Moreover, the pH of the container including solely Na^+^-resin ranged from 7.85 to 8.03 (Figure 9), meaning that the Na^+^-form resin did not affect the pH of the saltwater during the in vivo testing. These observations indicate that the sodium form of the resin is less hazardous to shellfish and probably less prone to interact with the ecological environment. Accordingly, we proceeded with the depuration experiments using the Na^+^-form resin. Although we observed a significant difference in the first 24 h (again due to decreasing dcSTX concentration), the acceleration observed in the presence of resin is very small compared to that verified without resin. In fact, after 48 h no significant differences were observed, indicating that, despite cancelling the effect of pH using the Na^+^-form resin, the clearance rate did not seem to improve. As previously explained, the adsorption mechanisms in natural waters are strongly affected by the conditions and constituents of the medium, which decreases the effectiveness of the method. These factors, allied to the mussels’ apparent inability to filter the resin, assume a dominating role that prevails over the cation exchange theory that is successfully applied in the treatment of purest water.

## 4. Conclusions

The development of detoxification methods for live bivalve molluscs has been attempted for a long time by several research groups. As far as we know, a truly effective and feasible methodology in a short period of time (e.g., 48 h) has not yet been found. Many of the studies carried out so far are restricted to aqueous solutions, without in vivo implementation, often leading to a false expectation of success. Based on the molecular properties of this group of toxins and considering the mode of adsorption through exchange of ions between chemical entities, we decided to explore the use of a cation-exchange resin (H-form and Na^+^-form) with the purpose of accelerating the detoxification process of shellfish. First, in vitro experiments were carried out using a culture of *G. catenatum* (one of the main dinoflagellate producers of paralyzing toxins). Then, the methodology was tested in vivo using mussels (*M. edulis*), which are known as sentinel organisms and are often used in this type of studies. In vitro studies using cultures of *G. catenatum* revealed around 80% decrease in overall toxicity upon exposure to the resin, after 48 h. Interestingly, the effect of molecular structure on the preferential adsorption of some toxins over others was evident. In this work, four main toxins (C1&2, dcSTX, GTX5, GTX6) were quantified, with dcSTX being responsible for the main decrease in toxicity, followed by GTX5. Differences were mainly attributed to steric hindrance, electronic effects, and magnitude of the overall positive charge. Although we have not identified here other analogues with high toxic potential (e.g., STX and NEO), considering the physicochemical properties of the molecules and the results obtained in this work and by other authors [38], it is expected that the adsorption of these toxins may occur successfully in solution using these cation-exchange resins. The in vivo experiments did not show evidence of a significant acceleration of the clearance process, compared to resin-free clearance. This could be due to competition from other natural substances (e.g., salts, organic matter) for the same binding sites, interactions between molecules, increasing steric hindrance and/or blocking pores, or difficulties in resin absorption by the mussel. This methodology could be promising in detoxification, but it is not yet ready to be applied. Therefore, further adjustments to the proposed methodology, such as changing the functional groups to tailor the system’s physicochemical properties, are needed in follow-up work. Nevertheless, we believe that our unprecedented approach and the findings here disclosed are relevant, in that they draw attention to the importance of testing methodologies in vivo and they reveal some conditions that may strongly affect the experimental results, namely the pH-effect and its potential consequence on the metabolism and well-being of mussels. Additionally, in vivo experiments have demonstrated the occurrence of bioconversions between toxin molecules. Based on the information obtained, the production of dcSTX from GC3 (by hydrolysis) is proposed, as well as the conversion of C1&2 into GTX5.

## Figures and Tables

**Table 1:** PST concentrations, in µM (average ± standard deviation, SD), in *G. catenatum* cultures during the removal studies using the H-form resin. At time 0 and 48 h control, no resin was present.

Toxin	T0 (0 h)	T1 (1 h)	T2 (3 h)	T3 (19 h)	T4 (26 h)	T5 (48 h)	C (48 h)	Removal (48 h, %)
C1,2	0.24 ± 0.01	0.25 ± 0.01	0.24 ± 0.00	0.25 ± 0.01	0.26 ± 0.01	0.25 ± 0.00	0.25 ± 0.01	---
DcSTX *	0.33 ± 0.00	0.14 ± 0.15	0.12 ± 0.13	0.06 ± 0.11	ND	ND	0.33 ± 0.01	100 ± 0
GTX5	0.09 ± 0.00	0.06 ± 0.00	0.06 ± 0.01	0.09 ± 0.00	0.06 ± 0.02	0.05 ± 0.02	0.09 ± 0.00	55 ± 5
GTX6	0.57 ± 0.02	0.58 ± 0.01	0.56 ± 0.01	0.58 ± 0.02	0.57 ± 0.01	0.56 ± 0.02	0.57 ± 0.03	---

* For the calculation of the average and standard deviation, the value of zero was assigned when the toxins were not detected.

**Table 2:** PST concentrations, in µg STX.2HCl eqv/Kg (average ± SD), in mussels *Mytilus edulis* obtained from in vivo studies, in the absence of resin or in the presence of the H-form (2nd set) and Na^+^-form (3rd set) resins. At time 0 (day 6 of each experiment), no resin was present.

		2nd Set of Experiments						3rd Set of Experiments				
		Without Resin			With H-Form Resin				Without Resin		With Na^+^-Form Resin	
	0 h	24 h	48 h	70 h	24 h	48 h	70 h	0 h	24 h	48 h	24 h	48 h
C1&2	23 ± 10	17 ± 7	13 ± 3	14 ± 4	10 ± 2	14 ± 5	21 ± 4	28 ± 1	24 ± 2	18 ± 1	21 ± 2	19 ± 0
dcSTX	841 ± 113	598 ± 73	567 ± 71	563 ± 73	505 ± 36	540 ± 67	627 ± 106	853 ± 10	782 ± 32	703 ± 23	711 ± 19	686 ± 9
GTX5	18 ± 4	15 ± 4	13 ± 2	14 ± 4	11 ± 1	13 ± 3	19 ± 3	18 ± 1	16 ± 1	14 ± 1	15 ± 1	16 ± 0
GTX6	87 ± 1	83 ± 1	79 ± 1	82 ± 2	81 ± 1	78 ± 1	84 ± 2	82 ± 5	78 ± 0	80 ± 0	82 ± 5	79 ± 0
TOTAL	973 ± 138	728 ± 96	672 ± 78	673 ± 82	607 ± 40	645 ± 75	750 ± 114	981 ± 15	900 ± 34	815 ± 24	828 ± 17	799 ± 11
Reduct. (%)	---	28 ± 9	33 ± 11	33 ± 11	34 ± 7	31 ± 6	19 ± 10	---	8 ± 4	16 ± 3	16 ± 4	19 ± 3

## Data Availability

Data is contained within the article and Supplementary Materials.

## Supplementary Materials

The following supporting information can be downloaded at: https://www.mdpi.com/article/10.3390/foods12040768/s1, Figure S1: Chromatograms of a chemical blank oxidized by peroxide and periodate; Figure S2: Chromatograms of mussels’ samples (*Mytilus edulis*), containing or not PST, not oxidized (procedure as in periodate oxidation, with matrix modifier); Figure S3: Chromatograms of matrix modifier (prepared from oysters *Crassostrea gigas*), oxidised with periodate (PST-free sample); Figure S4: Results of the PST clearance tests in live mussels (without resin and with H-form resin); Figure S5: Results of the PST clearance tests in live mussels (without resin and with Na^+^-form resin); Figure S6: Experimental design for the study of the pH-effect in in vivo experiments. Table S1: Average size (length x width, in cm) of mussels *Mytilus edulis* used in the second set of experiments; Table S2: Average size (length x width, in cm) of mussels *Mytilus edulis* used in the third set of experiments; Table S3: Method parameters: determination coefficient (R^2^), detection limit (LOD), quantification limit (LOQ); Table S4: PST concentration, estimation in µg STX.2HCl eqv/Kg (average ± SD), in *G. catenatum* cultures, during removal studies using the H-form resin.
