# Supplementary material for: Cation-Exchange Resin Applied to Paralytic Shellfish Toxins Depuration from Bivalves Exposed to Gymnodinium catenatum
Source: Foods. 2023 Feb 10;12(4):768. doi: 10.3390/foods12040768 (PMC9955756; doi:10.3390/foods12040768)
Supplement: Supplementary file 1 [file foods-12-00768-s001.zip › foods-2199879-supplementary.pdf]

Supporting information

Cation-exchange resin applied to Paralytic shellfish toxins depuration from bivalves exposed to *Gymnodinium catenatum*

Joana F. Leal[1], Gabriel Bombo[2], Patrícia S. M. Amado[1], Hugo Pereira[2], Maria L. S. Cristiano[1*]

[1] *Centre of Marine Sciences (CCMAR) and Department of Chemistry and Pharmacy, Faculty of Science and Technology – University of Algarve, Campus de Gambelas 8005-139 Faro, Portugal*

[2] *GreenCoLab – Associação Oceano Verde, Universidade do Algarve, Campus de Gambelas, 8005-139 Faro, Portugal*

Materials and methods

Table S1 – Average size (length x width, in cm) of mussels Mytilus edulis used in the second set of experiments. The values inside the parentheses correspond to the standard deviation.

Tank 1	Tank 2	Tank 3	Tank 4	Tank 5	Tank 6
6.4(0.7) x 3.8(0.4)	6.2(0.7) x 4.0(0.5)	6.2(0.6) x 3.8(0.5)	6.4(0.7) x 4.0(0.5)	6.6(0.9) x 4.4(0.9)	6.4(0.6) x 3.9(0.6)

Table S2 – Average size (length x width, in cm) of mussels Mytilus edulis used in the third set of experiments. The values inside the parentheses correspond to the standard deviation.

Tank 1	Tank 2	Tank 3	Tank 4
6.6(0.5) x 3.5(0.5)	6.6(0.5) x 3.7(0.3)	6.6(0.4) x 3.6(0.3)	6.6(0.5) x 3.6(0.4)

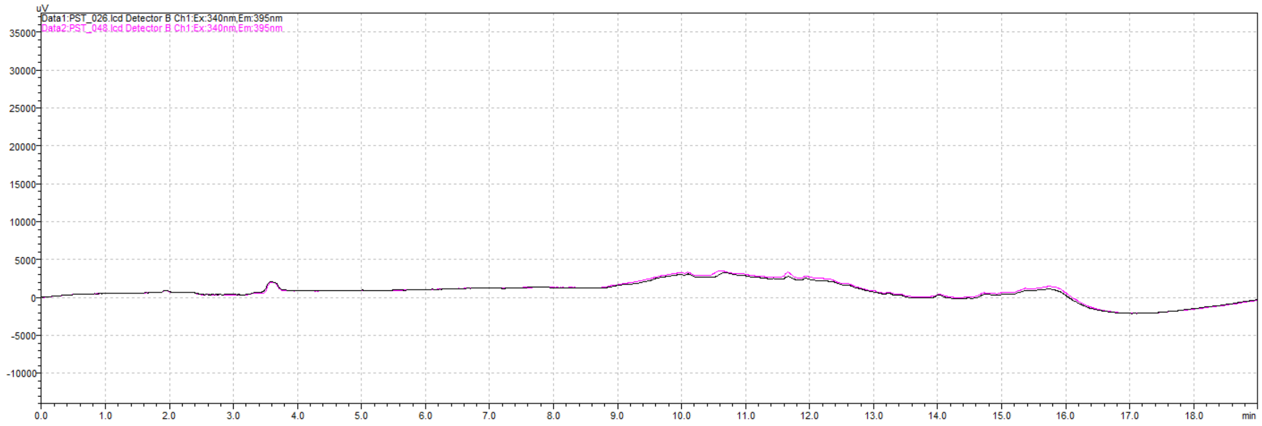

Figure S1 – Chromatograms of a chemical blank oxidized by peroxide (black) and periodate (pink)

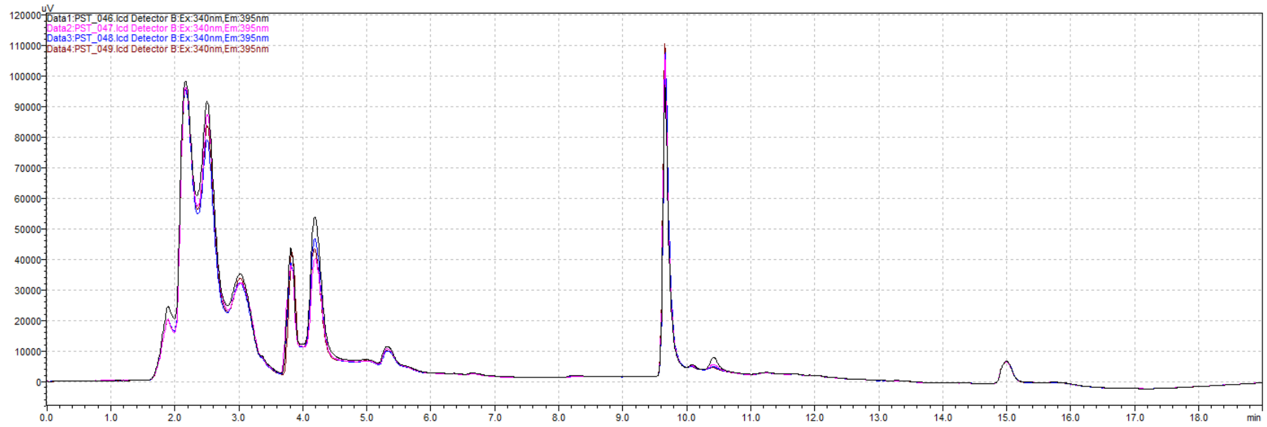

Figure S2 – Chromatograms of mussels' samples (Mytilus edulis) after SPE-C18, containing or not PST, not oxidized (procedure as in periodate oxidation, with matrix modifier). The products shown are naturally fluorescent co-extractives.

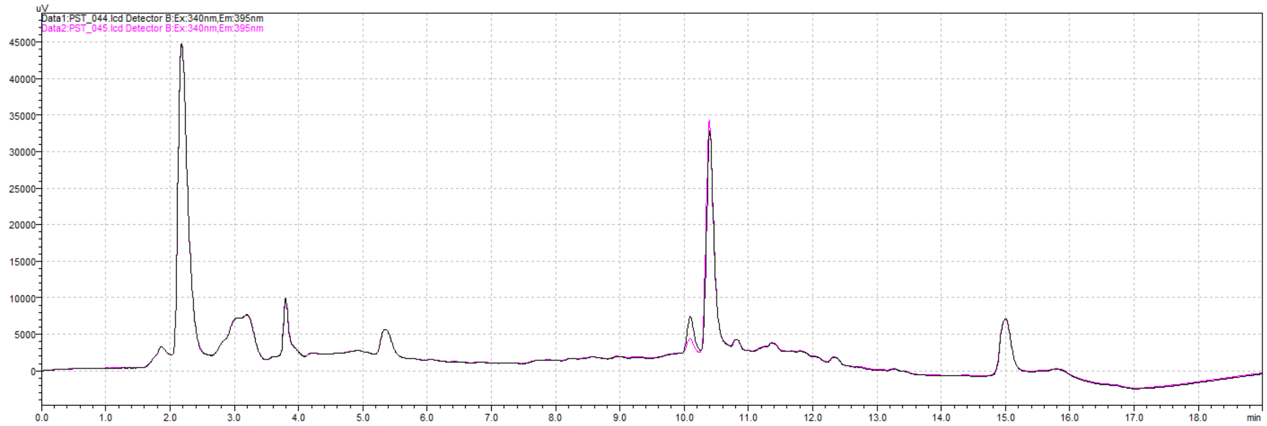

Figure S3 – Chromatograms of matrix modifier (prepared from oysters Crassostrea gigas), oxidised with periodate (PST-free sample).

Results

➢ HPLC-FLD analysis

Table S2 – Method parameters: determination coefficient (R^2), detection limit (LOD), quantification limit (LOQ).

Toxin	R^2	LOD (µM)	LOD (µg STX.2HCl eqv/kg) *	LOQ (µM)	LOQ (µg STX.2HCl eqv/kg) *
dcGTX2&3	≥ 0.9998	≥ 0.005	≥ 6	≥ 0.02	≥ 21
C1&2	≥ 0.9999	≥ 0.003	≥ 1	≥ 0.01	≥ 3
dcSTX	≥ 0.9993	≥ 0.02	≥ 63	≥ 0.07	≥ 209
GTX2&3	≥ 0.9996	≥ 0.01	≥ 25	≥ 0.05	≥ 84
GTX5	≥ 0.9999	≥ 0.005	≥ 1	≥ 0.02	≥ 4
STX	≥ 0.9981	≥ 0.01	≥ 34	≥ 0.04	≥ 112
GTX1&4	≥ 0.9990	≥ 0.03	≥ 169	≥ 0.09	≥ 565
NEO	≥ 0.9993	≥ 0.02 (0.05) †	≥ 184	≥ 0.08 (0.16) †	≥ 615
C3&4	≥ 0.9994	≥ 0.01 (0.03) †	≥ 11	≥ 0.04 (0.12) †	≥ 36
GTX6	≥ 0.9992	≥ 0.02	≥ 13	≥ 0.07	≥ 43
dcNEO	≥ 0.9984	≥ 0.03 (0.05) †	≥ 97	≥ 0.11 (0.18) †	≥ 322

† For the toxins C3&4, NEO and dcNEO, the values between parenthesis correspond to the different values obtained from calibration curves constructed without matrix modifier (data only used in samples from *G. catenatum* cultures).

* The conversion of the values in µM to µg STX.2HCl eqv/Kg was performed according to the following equation:

$$C_i(\mu g\ STX.2HCl\ eqv/Kg) = C_i(\mu M) \times TEF \times MW\ (g/mol) \times \frac{V_E(mL)}{m_H(g)} \times D_f \qquad \text{(Eq. S1)}$$

Where C_i is the concentration of each toxin; TEF is the toxicity equivalence factor for each toxin, according to EFSA; MW is the molecular weight of saxitoxin dihydrochloride (372.2 g/mol); V_E is the volume after the extraction (10 mL); m_H is the mussels' homogenised tissue (5.0 g); D_f is the dilution factor for each toxin throughout the procedure.

➢ **PST clearance in bivalves:**

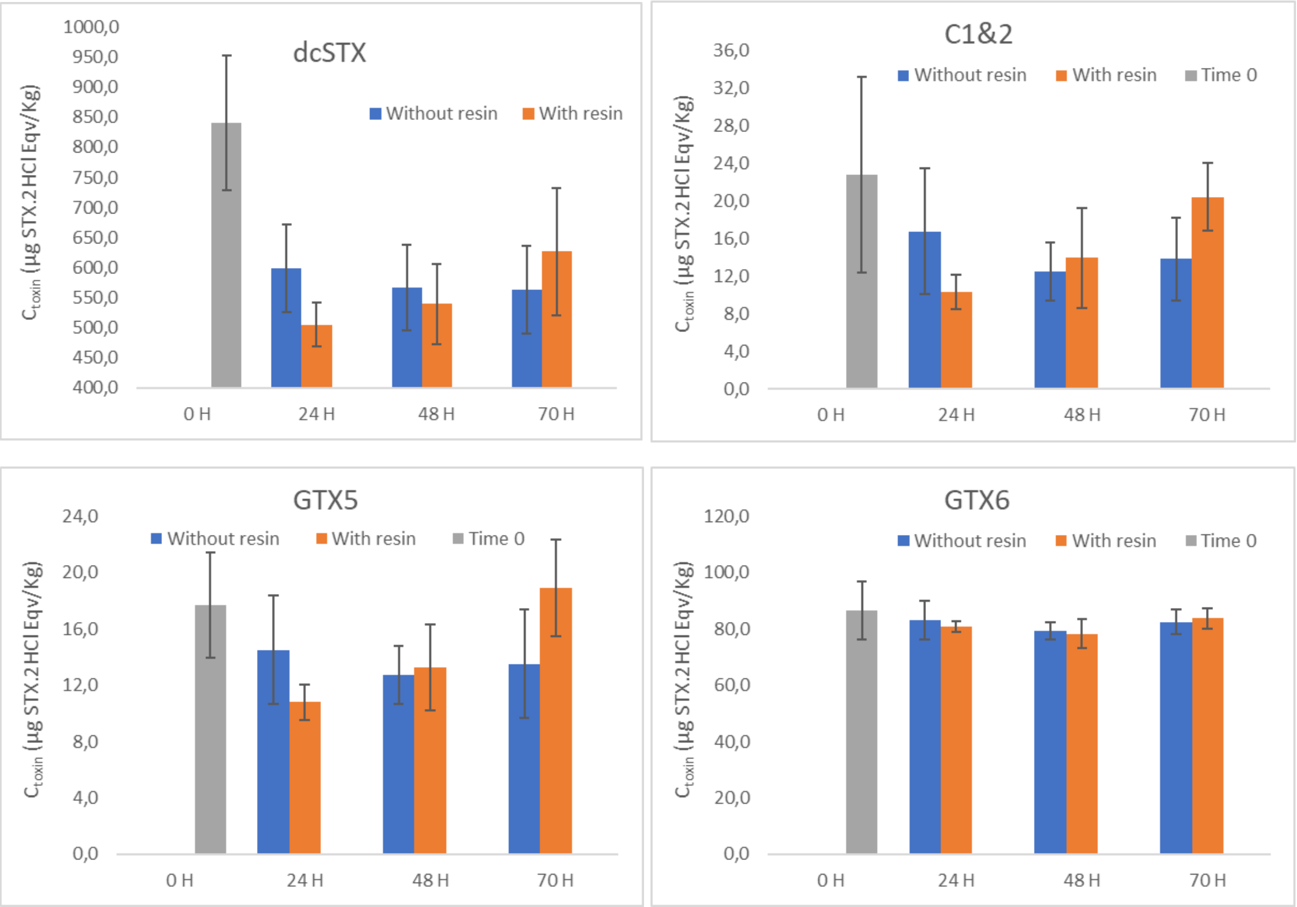

*Figure S4 – Results of the PST clearance tests in live mussels (without resin and **with H-form resin**).*

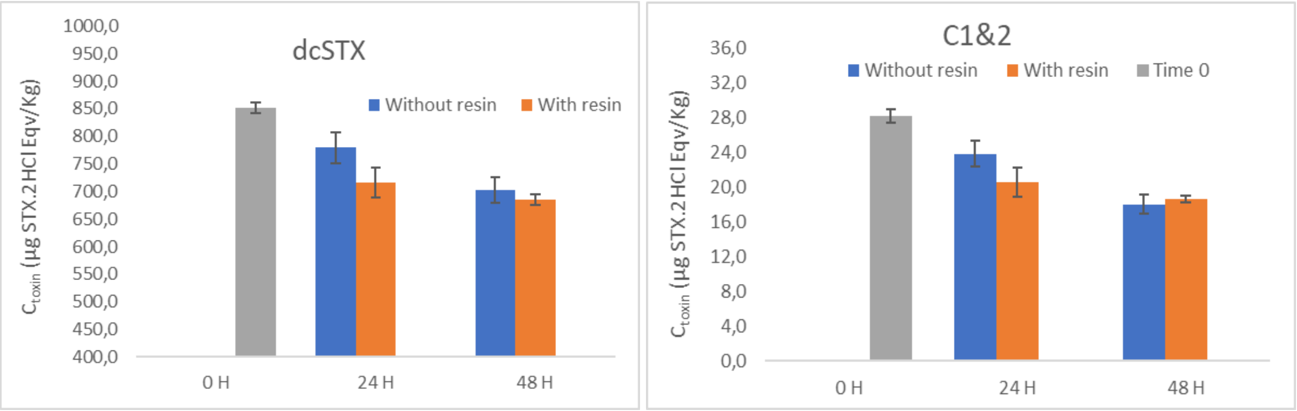

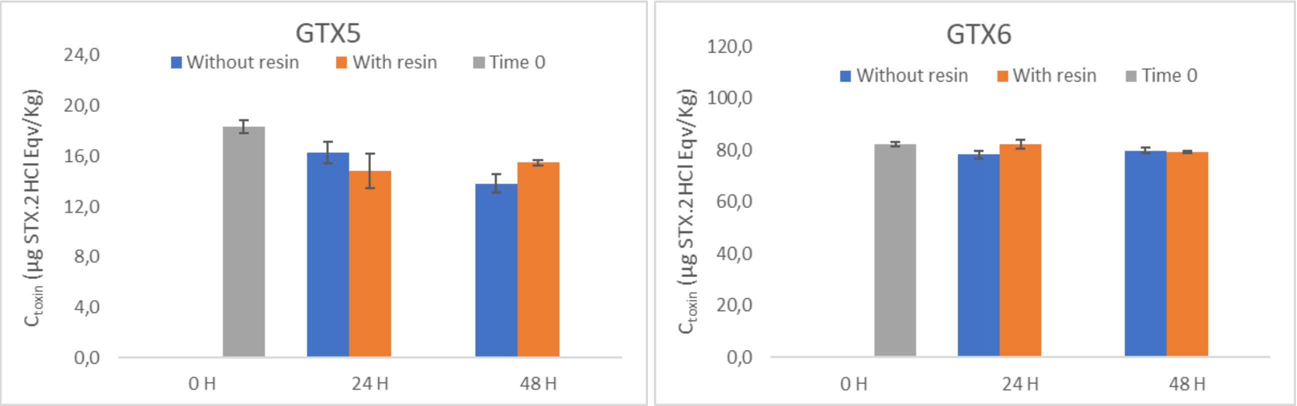

*Figure S5 – Results of the PST clearance tests in live mussels (without resin and **with Na$^+$-form resin**).*

➢ PST concentrations

Table S4 – PST concentration, estimation in µg STX.2HCl eqv/Kg (average ± SD), in G. catenatum cultures, during removal studies using the H-form resin. At time 0 and 48-h control, no resin was present. Conversion from concentrations in µM were done using equation S1.

Toxin	T0 (0 h)	T1 (1 h)	T2 (3 h)	T3 (19 h)	T4 (26 h)	T5 (48 h)	C (48 h)
**C1,2**	18.2 ± 0.9	18.6 ± 0.5	17.6 ± 0.2	18.5 ± 0.9	19.3 ± 0.4	18.7 ± 0.3	18.4 ± 0.9
**dcSTX***	249.3 ± 1.2	104.6 ± 109.4	92.0 ± 96.1	45.9 ± 83.0	ND	ND	248.6 ± 4.5
**GTX5**	6.7 ± 0.1	4.6 ± 0.3	4.7 ± 0.9	6.6 ± 0.1	4.6 ± 1.2	4.0 ± 1.5	7.1 ± 0.2
**GTX6**	42.8 ± 1.2	42.9 ± 0.5	41.9 ± 0.8	43.2 ± 1.2	42.6 ± 0.9	41.6 ± 1.2	42.7 ± 2.2

* For the calculation of the average and standard deviation, the value of zero was assigned when the toxins were not detected.

➢ H-form *vs.* Na$^+$-form resins

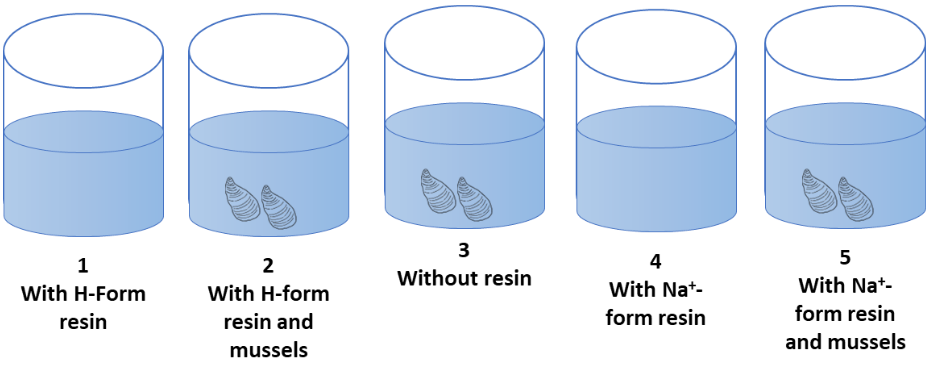

Figure S6 – Experimental design for the study of the pH-effect in in vivo experiments.
